# A Chinese version of the Language Screening Test (CLAST) for early-stage stroke patients

**DOI:** 10.1371/journal.pone.0196646

**Published:** 2018-05-04

**Authors:** Hongyan Yang, Shenghua Tian, Constance Flamand-Roze, Ling Gao, Wei Zhang, Yan Li, Jiajia Wang, Zhou Sun, Ying Su, Libin Zhao, Zhihou Liang

**Affiliations:** 1 Department of Neurology, Union Hospital, Tongji Medical College, Huazhong Science & Technology University, Wuhan, Hubei, China; 2 Department of Endocrinology, Union Hospital, Tongji Medical College, Huazhong Science & Technology University, Wuhan, Hubei, China; 3 Department of Neurology, Centre Hospitalier du Sud Francilien, Corbeil-Essonne, France; 4 Department of Neurology, First Affiliated Hospital of Shanxi Medical University, Taiyuan, Shanxi, China; 5 Department of Neurology, Luoyang Central hospital affiliated to Zhengzhou University, Luoyang, Henan, China; 6 Department of Neurology, Binzhou people’s hospital, Binzhou, Shandong, China; 7 Department of Anesthesia, Maternal & Child Health Hospital of Bao’an District, Shenzhen, Guangdong, China; Taipei Veterans General Hospital, TAIWAN

## Abstract

There is a severe lack of aphasia screening tools for bedside use in Chinese. A number of aphasia assessment tools have recently been developed abroad, but some of these scales were not suitable for patients with acute stroke. The Language Screening Test (which includes two parallel versions [a/b]) in French has been proven to be an effective and time-saving aphasia screening scale for early-stage stroke patients. Therefore, we worked out a Chinese version of the LAST taking into consideration Chinese language and culture. Two preliminary parallel versions (a/b) were tested on 154 patients with stroke at acute phase and 107 patients with stroke at non-acute phase, with the Western Aphasia Battery serving as a gold standard. The equivalence between the two parallel versions and the reliability/validity of each version were assessed. The median time to complete one preliminary Chinese version (each had some item redundancy) was 98 seconds. Two final parallel versions were established after adjustment/elimination of the redundant items and were found to be equivalent (intra-class correlation coefficient: 0.991). Internal consistency is(Cronbach α for each version [a/b] was 0.956 and 0.965, respectively) good. Internal validity was fine: (a) no floor or ceiling effect/item redundancy; (b) construct validity revealed a 1-dimension structure, just like the French version. The higher educated subjects scored higher than their lower educated counterparts (*p<0*.*01*). The external validity: at the optimum cut-off point where the score of version a/b <14 in higher educated group(<13 in lower): the specificity of each version was 0.878/0.902(1/1 in lower) and sensitivity was 0.972/0.944(0.944/0.944 in lower). Inter-rater equivalence (intra-class correlation coefficient) was 1. The Chinese version of the Language Screening Test was proved to be an efficient and time-saving bedside aphasia screening tool for stroke patients at acute phase and can be used by an average medical physician.

## Introduction

Aphasia represents one of the most common consequence of stroke[1], and affects about one-third of acute-phase stroke patients. It lowers quality of life, affects social interaction, and even induces depression[2]. Godecke et al demonstrated that a very early aphasia therapy for mild to severe aphasia of post-stroke patients proved to be significantly effective over short and medium term at follow-up (six months), and that the timing and intensity of aphasia therapy post-stroke were significantly associated with the recovery of communication ability[3–5]. Most well-established scales for aphasia diagnosis, such as the Western Aphasia Battery (WAB) and the Boston Diagnostic Aphasia Evaluation (BDAE), are too long to complete, especially during the acute phase of stroke, and must be administered by speech and language therapists[6–8]. A number of aphasia assessment tools for bedside use have recently been developed[9–17], but such scales remain unavailable in China. Moreover, some of the aforementioned scales have some limitations in terms of administration, such as requiring stimulus cards to be readily available[15,18]; Application of some tests is limited by the presence of visual field deficits, visual neglect or inattention, illiteracy, deafness.[15,18]; Some tests have no norms against which to interpret scores[11,19,20],and some scales only address particular expressive language skills such as semantic verbal fluency [17]; Moreover, some test have been proved to be insensitive [11].

The Language Screening Test (LAST) in French generated by Constance Flamand-Roze, which was designed for aphasia screening in emergency setting, avoids the aforementioned shortcomings and has been proved to be suitable for routine bedside evaluation of patients with acute stroke in clinical practice[9]. The released German version of the LAST has also been proved to be reliable and valid[21]. Therefore, LAST may has good language flexibility or compatibility and can be easily tailored into a different language version by making some slight modifications according to a certain linguistic and cultural context. Thus, we generated a Chinese version of the LAST (CLAST) by taking into account Chinese language and culture, and tested the CLAST in post-stroke patients from several areas geographically located in Henan, Shandong, Shanxi, and Hubei provinces, with an attempt to develop an efficient and time-saving bedside aphasia screening tool for Chinese-speaking patients suffering from acute-phase stroke worldwide.

## Methods

The study was approved by the Clinical Trial Ethics Committee of Huazhong University of Science and Technology, Wuhan, China. (Approval NO. S102). Verbal informed consent was obtained from all recruited subjects or their legally acceptable representatives prior to the experiment.

### Development of the CLAST (Key elements of study design)

#### Features of the LAST

To avoid retest bias of the LAST, two parallel versions were constructed. Each item on the two scales was different (apart from the automatic speech item) but strictly matched. Each version of the LAST consisted of 5 subtests and a total of 15 items. One (1) point was awarded for correct answers given within 5 s, otherwise the score was zero (0). The maximum score was 15, covering two sub-scores, namely, an expression index (including naming, repetition, and automatic speech; the "maximum " score was 8) and a receptive index (including picture recognition and verbal instructions; the "maximum" score was 7). The sub-items are detailed under the following subheading.

#### Modifications of sub-items(similarities and differences between LAST and initial CLAST)

To help us achieve better accuracy in the scale adaptation, the French authors of LAST gave us a great many help, including translation and back-translation and cross-cultural adaptation of the items. By referring to the design principles of the LAST[9], and considering the difference in everyday familiarity (subjective verbal frequency) between Chinese and French[22,23], we made some slight modifications as follows. The different versions CLAST are provided in Appendices(S1–S3 Files).

Our CLAST also consists of five subtests, but some modifications were made: (1) in naming of five black-and-white drawings, considering the cultural difference between Chinese and French, “dinner knife” in LAST-b was replaced by “chopsticks” with nearby verbal frequency in CLAST-b; (2) in oral repetition, to keep the same number of syllables with LAST, some small adjustments, such as reducing function words, were made according to syntactic rules of Chinese. As a result, we came up with one word with 4 syllables and one sentence with 11 syllables with semantically consistent with LAST; (3) in automatic speech, i.e. counting from 1 to 10; sentences were left semantically unchanged in LAST. (4) in picture recognition, complex items replacements were made given that distractors were more complicated in Chinese, the replaced items are detailed in the next paragraph; (5) in execution of three verbal commands (simple, semicomplex,complex), sentences were also left semantically unchanged in LAST.

Details of picture-recognition: in LAST, four orally presented target words were depicted within a set of eight pictures, with the four distractor pictures being either visually, semantically, phonologically or visually related to one of the target words, complex item replcements were made considering that distractors are more complicated in Chinese. In the CLAST, the semantic and visual pairs of distractors in the LAST were adopted and were left semantically unchanged. Nonetheless, the distractors are more complicated in the CLAST than in the LAST since in Chinese language phonological and semantic distractions are interwoven and/or concomitant. The replacements were selected from common objects based on frequencies of Chinese words and characters [23]. For example, in the LAST-a “lapin” (rabbit) and it’s phonological distractors “pin” (pine) were replaced by “Songshǔ” (squirrel) and it’s interwoven phonological and semantic distractors “Songshù” (pine); Similar, “cuillère” (spoon) and “lait caillé” (cheese) were replaced by “Yiguì” (wardrobe) and “Yijià” (hanger), in the LAST-b, “chapeau” (cap) and “gateau” (cake) were replaced by “bàozhi” (newspapers) and “bāozi” (steamed stuffed bun); and “main” (hand) and “pain” (bread) were replaced by “shuǐtǒng” (bucket) and “shuǐhú” (kettle). To avoid possible failure, we prepared a backup pair of semantic distractors to the “Picture recognition” subtest of each version, but it was end up by discarding the reserves for item redundancy. As a result, the “Picture recognition” subtest of our initial CLAST contained five items (with five corresponding distractors). The replaced pictures in the CLAST were drawn by the same artist of LAST to ensure the consistency of the style. The comparsion of distractors between LAST and CLAST are included as Appendices(S6 File).

### Patients and instruments

To verify the reliability and validity of CLAST, we enrolled stroke patients at “acute-phase” and “non-acute phase”. The consecutive “acute-phase” patients included were from the departments of neurology of four general hospitals: Union Hospital of Tongji Medical College, Huazhong Science & Technology University; the First Hospital of Shanxi Medical University; Luoyang Central Hospital Affiliated with Zhengzhou University; and Binzhou People’s Hospital with time frame lasting from August to October in 2014.

Patients at “non-acute phase” were divided to an aphasic group and a non-aphasic group with the WAB serving as a gold standard[6]. Considering the short hospitalization time and the feasibility to complete the test of the WAB of the stroke patients, patients who were 10 days after stroke onset was defined as the patients at “Non-acute” phase in our test. Non-aphasia patients were enrolled from August to October, and their aphasia counterparts were from August to December in 2014.

The demographic information of the participants, including educational background, together with clinical diagnosis, including imaging findings, was collected.

#### Inclusion criteria

Patients with Mandarin as their native language and satisfied the following criteria were included:(1) within 3 days after stroke onset (acute-phase patients) and (2) had stroke attacks 10 days’ later(including those who had stroke episodes a few years ago) and was able to complete the WAB (patients with stroke at non-acute phase). Stroke was confirmed by brain radiological imaging(Computed Tomography or Magnetic Resonance Imaging).

#### Exclusion criteria

Patients were excluded from study if they met any of the following: (1) being mentally retarded or demented premorbidly at the time of study or before this stroke episode; or having (2)psychiatric disorders;(3)visual problems;(4)auditory problems;(5)consciousness disorders(score of Glasgow Coma Scale (GCS) <15);(6)native language was not Mandarin.

### Procedures

We tested the CLAST in post-stroke patients from several areas geographically located in Henan, Shandong, Shanxi, and Hubei provinces. Both the CLAST-a and CLAST-b were used in all patients. Nine examiners were involved in our test: three examiners worked forUnion Hospital of Tongji Medical College, Huazhong Science & Technology University; the other six were from the First Hospital of Shanxi Medical University, Luoyang Central Hospital, and Binzhou People’s Hospital, with two coming from each hospital.

#### Test procedure in the acute-phase patients

CLAST-a and CLAST-b was administered alternately as the first test for the consecutive patients. During the interval between the two tests, the subjects were evaluated with the National Institutes of Health Stroke Scale (NIHSS). To assess inter-rater reliability, during the the first test(by CLAST-a or CLAST-b), two examiners rated the patients at bedside(a doctor who had received professional training in conducting a number of aphasia scales such as WAB, BDAE,CLAST, etc; and a resident who was randomly selected from students on a standardized training program, given a 5-minute training session for the test). One of the examiners asked the patient questions according to the first test(by CLAST-a or CLAST-b, namely, version-a or version-b of CLAST), meanwhile, the two examiners made their judgment independently, and the amount of time taken to finish the test was recorded. Following an interval specified by NIHSS, the other version-b/a of CLAST was administered by the examiner who hadn’t questioned the patients in the first round.

#### Test procedure in the non-acute stroke patients

Participants were first given the WAB to determine if they had aphasia by one examiner, who was blinded to the site of stroke lesion[6,24]. Afterwards, the CLAST-a and CLAST-b and NIHSS were administered in the same way they were given to the acute-phase patients(only one examiner accomplished that procedure). To reduce potential bias of assessment, the rating of the WAB was undertaken by one examiner who had received professional training in conducting a number of aphasia scales such as WAB, BDAE, CLAST, etc. The WAB and CLAST were administered by two different examiners who were blind to the results of the other examiner, and all the examiners were blind to the sites of stroke lesion.

**Fig 1:**
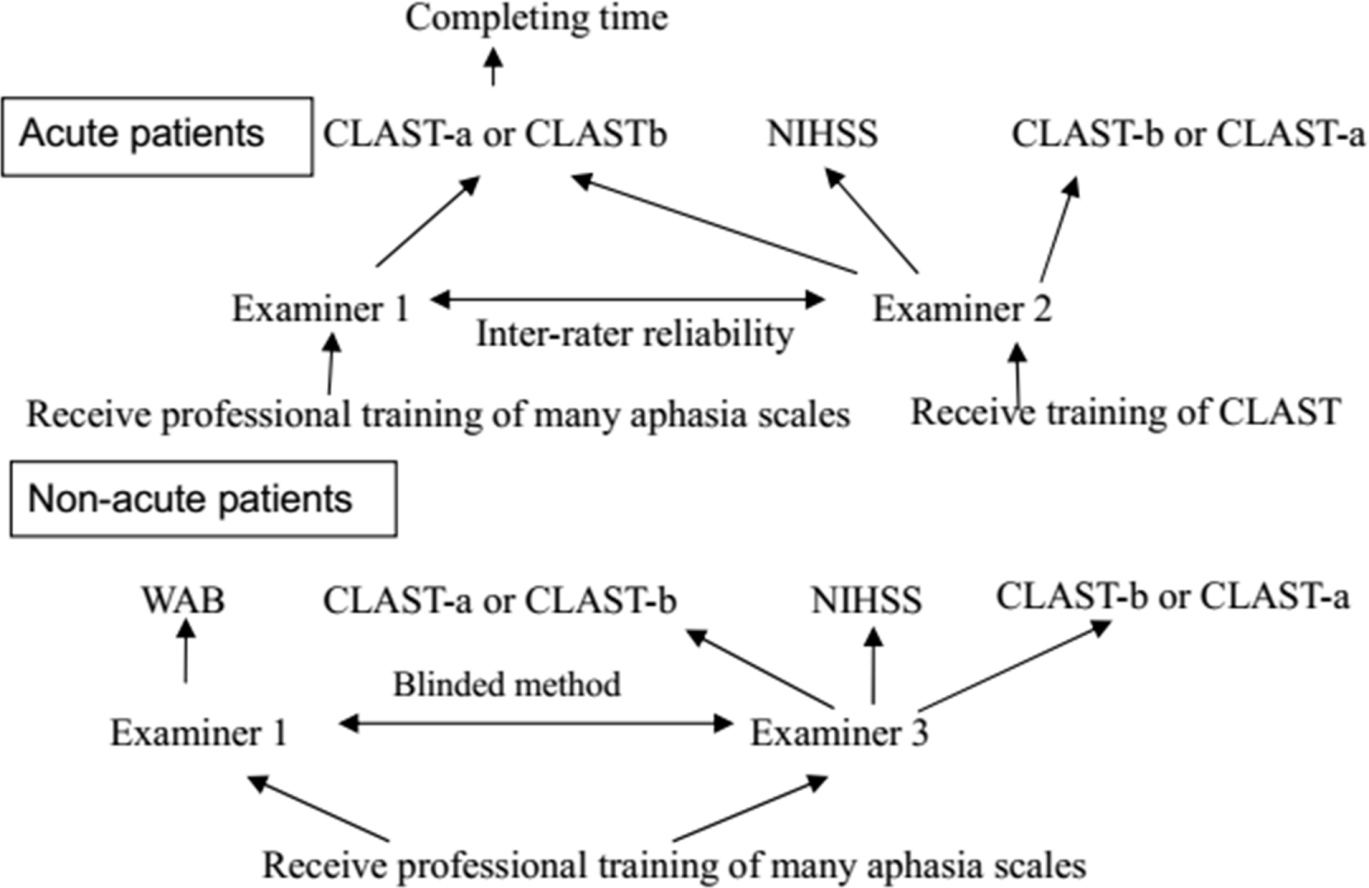
The flow chart to administer aphasia scales in acute and non-acute stroke patients.

The flow-chart of the above-mentioned tests in all patients is given in Fig 1 below.

### Validation of the CLAST

**Fig 2:**
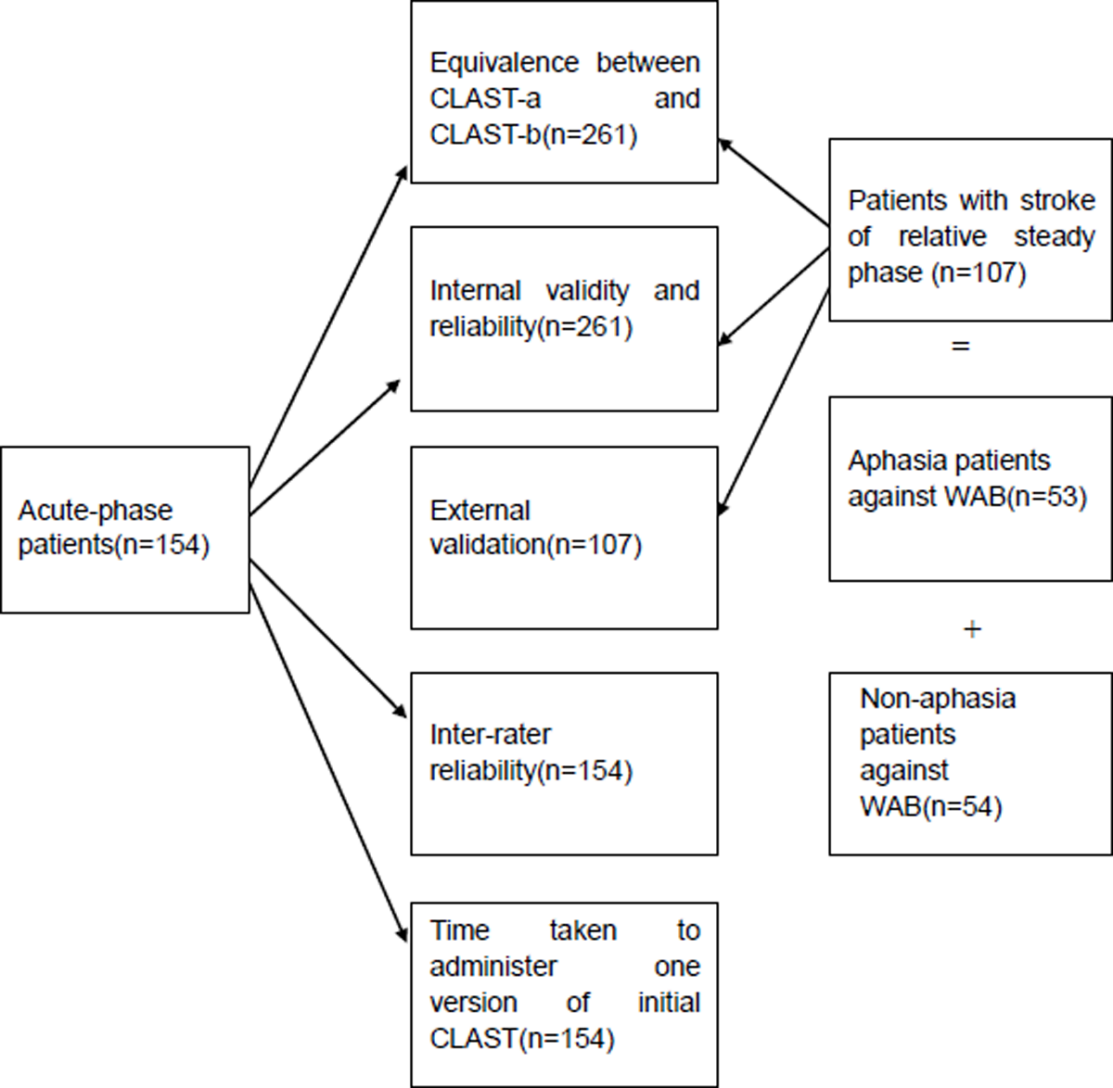
Schematic representation of validation process of Chinese version of the Language Screening Test (CLAST). Please note that the floor and ceiling effects were assessed in aphasia group, and discrimination validity was assessed in the non-acute group.

The CLAST was validated in terms of the following aspects: the equivalence between the two versions, the internal validity of the two versions (including item redundancy, ceiling and floor effect, construct validity, and discrimination validity). External validity against the WAB and reliability (including internal consistency and inter-rater reliability), the relation between CLAST scores and educational background. A schematic representation of the study design is shown in Fig 2.

### Statistical analysis

The equivalence between the two versions of the CLAST was assessed by calculating the intra-class correlation coefficient (ICC) from the two total scores and parallel items[25]. Internal validity was assessed in terms of four aspects. First, the Spearman correlation matrix was used to detect item redundancy[26]. Second, the ceiling or floor effect was detected. The floor and ceiling effects, the percentages of the sample scoring the minimum and maximum possible scores, respectively, reflect the extent that scores cluster at the bottom and top of the scale range. Floor and ceiling effects <20% were considered to be significant. Third, construct validity was determined by utilizing exploratory factor analysis[27]. Factor analysis was carried out as follows. General Least Squares were used to extract factors. Oblique rotations were adopted in order to detect the correlations between the extracted factors. We protocol to use the scree test to determining the most appropriate number of factors to retain. Then, the Mann-Whitney U test was employed to compare the score of aphasia patients with their non-aphasia counterparts to verify discrimination validity(It was assumed that non-aphasia patients would have higher scores than aphasia patients, *p*<0.01). We computed Cronbach α to obtain the internal consistency. The ICC was used to represent inter-rater reliability. The external validity was obtained by plotting the receiver operating characteristic (ROC) curve, and the area under the ROC curve was used to indicate the diagnostic accuracy of the CLAST. The closer the ROC curve to the upper left-hand corner, the higher the overall accuracy of the test[28]. The Mann-Whitney U test was utilized to compare the performance of the two groups of different education levels (higher-educated patients who received junior middle school education and above *vs*. lower-educated patients who received elementary education and below) to understand the relationship between CLAST scores and educational background.

SPSS19.0 software package was used for all the statistical analyses.

## Results

### Sample description

Acute-phase patients: One hundred sixty seven consecutive patients were admitted to the neurology departments for suspected acute stroke during a 5-month period. Thirteen patients were excluded (severe consciousness impairment [n = 5], blindness [n = 3], history of dementia [n = 2], deafness [n = 1], psychiatric disorders [n = 1], refusal to participate [n = 1]). The remaining 154 acute patients were included. Their post-stroke time range from 1 to 3 day(s),the average and median time being 1.95 and 2 days respectively. Their mean age was 60.21 years (range: 24 to 88 years). Of them, 112 were males. Most of the patients (90.3%) had cerebral infarction. Their median NIHSS score was 3.00 (interquartile range: 4.25). The distribution of their educational levels is shown in Table 1.

**Table 1:** Baseline data of all patients, including the educational level of all patients(n = 261) and lesion sites of the non-acute patients(n = 107).

	Age(mean±SD)	Education(n/%)				
Patients(n = 261)	58.5±12.2	a	b	c	d	f
-Acute patients(n = 154)	60.2±12.3	14(9%)	30(20%)	42(27%)	43(28%)	25(16%)
-Non-acute patients(n = 107)	56.0±11.6	24(22%)	21(20%)	32(30%)	24(22%)	6(6%)
Non-acute patients(n = 107)		Lesion-site(n/%)				
Group		A	B	C	D	
-Aphasia(n = 53)	57.7±11.4	49(92%)	3(6%)	0(0%)	1(2%)	
-Non-aphasia(n = 54)	54.4 ±11.7	16(30%)	24(44%)	11(10%)	3(6%)	

We only recorded the lesion sites of the non-acute patients, exclusive of the patients in the acute phase. a: Tertiary degrees or above; b: High school or technical secondary school; c: Junior middle school; d: Elementary school; e: Illiterate. A: Left hemisphere; B: Right hemisphere; C: Cerebellum/brainstem; D: Bilateral hemisphere.

Non-acute phase patients: This group consisted of 107 patients hospitalized at Wuhan Union Hospital of Tongji Medical College, Huazhong Science & Technology University, including 53 aphasia patients and 54 non-aphasia patients, diagnosed against the Western Aphasia Battery. Their mean age was 56.03 years, ranging from 24 to 79. Most patients (88.8%) had cerebral infarction. The aphasia group had 40 males and 13 females (mean age: 57.72 years and range: 25 to 76), while the non-aphasia group included 43 males and 11 females (mean age: 54.37 years, and range: 24 to 79). In the non-acute group, the 107 patients were recruited, with their post-stroke time ranging from 10 to 1147 days after the stroke onset, and the average and median durations being respectively 65.6 and 14 days. The baseline data of the acute phase and non-acute phase patients with stroke are presented in Table 1.

### Statement

When we detected the item redundancy of initial CLAST by using Spearman’s correlation matrix, we found some item redundancies both in CLAST-a and CLAST-b(details are given insupplementary data,S4 File- The item redundancies in initial CLAST). Then these redundant items were removed and swapped to give a 14 item version of the CLAST (Final CLAST), details are listed in supplementary data S5 File -The adjustment of items in initial CLAST to overcome item redundancy. The data of the final CLAST could be obtained by “exchanging” scores of the paralleled items and “deleting” the scores of removed items from the data of initial CLAST, So the data of initial and final CLAST were both obtained from the original 261 patients, and no reassessment was conductedon the final CLAST. The final CLAST was then re-validated in the original subjects as follows (the Chinese and English version of the final CLAST are appended in the supporting information, S1–S3 Files).

### Statistical results

#### Time taken to complete the initial CLAST

All participants underwent the initial 16 item version of the CLAST, which took 98 seconds(median time) to administer. The time taken to complete 16 items lasted from 45 to 196 seconds and the interquartile range was 55 seconds.

#### Internal validity of the final CLAST

Spearman coefficients ranged from 0.3 to 0.9, suggesting that there was no item redundancy in the final CLAST. In terms of the floor and ceiling effects, in the 53 patients with aphasia enrolled, the percentages of the sample scoring the maximum were respectively 0% and 1.9% in CLAST-a and CLAST-b, and the percentages of the sample scoring the minimum were 7.5% and 5.7%, demonstrating that there was neither a floor nor a ceiling effect with both versions. Factor analysis (by General Least Squares, Oblique rotations and the scree test as aforementioned) revealed a two-factor solution, the two factors, with both eigenvalues above 1.0, explained variance of 70.860% in CLAST-a(74.261% in CLAST-b), but the two factors were highly correlated with a correlation coefficient of 0.624 in CLAST-a(0.762 in CLAST-b). Hence, we tried to merge the two related factors, namely, we limited the number of extracted factor to 1 with each version. Then, the sole factor accounted for 63.564% of the explained variance in CLAST-a (67.532% in CLAST-b),the factor loading of the 14 items of CLAST-a ranged from 0.488 to 0.930(and from 0.678 to 0.964 in CLAST-b), just as the acknowledged view: with high(0.80 and above) and mid-range factor loadings (0.40–0.60)[29]. Table 2 shows the loading of each item on the factor of each CLAST version. So, the CLAST, similar to the French LAST, can be said to reveal 1-dimensional structure. The Mann-Whitney U test showed that non-aphasia patients outperformed their aphasia counterparts with both versions (The sum of ranks in the non-aphasia group were 4310/4300 in CLAST-a/b against 1468/1478 in the aphasia group, p = 0.000<0.01 with both CLAST-a and CLAST-b), and discrimination validity was good.

**Table 2:** Factor loading details of the 14 items with each version of CLAST. Rotated component matrix using the Generalized Least Squares analysis, limited the number of extracted factor to 1 in each version and Oblique rotations (n = 261).

Variables	Factor	Variables	Factor
CLAST-a	1	CLAST-b	1
Telep	0.893	Penc	0.787
Pen	0.752	Telev	0.862
Pine	0.796	Fork	0.883
Croc	0.488	Gira	0.762
Chop	0.930	Butt	0.964
Math	0.872	Lite	0.855
The	0.627	Vaca	0.678
Auto	0.928	Auto	0.949
Squi	0.741	News	0.793
Armo	0.790	Pail	0.793
Ciga	0.720	Car	0.832
Eye	0.835	Toma	0.722
Don't d	0.866	Don't b	0.864
Put	0.803	Touc	0.702

The items in CLAST were presented as the above short form, their original form were detailed as follows. Variables in CLAST-a: *Telep-Telephone; Pen-Pen; Pine-Pineapple; Croc-Crocodile; Chop-Chopsticks; Math-Mathematics; The-The post man brings a letter to my neighbor; Auto-Automatic speech; Squi-Squirrel; Armo-Armoire; Ciga-Cigarette; Eye-Eye; Don't d-Don't take the drinking-class but the pen; Put-Put a hand on your head*, *then a finger on your nose*. Variables in CLAST-b: *Penc-Pencil; Telev-Television;Fork-Fork; Gira-Giraffe; Butt-Butterfly; Lite-Literature; Vaca-Vacationers would like strawberry ce-cream;Auto-Automatic speech; News-Newspaper; Pail-Pail; Car-Car; Toma-Tomato; Don't b-Don't take the book but the keys; Touc-Touch one of yours ears with one finger*, *then your foerhead with two fingers*.

#### Reliability of the CLAST

The internal consistency of the 14 items was good, with a Cronbach α of 0.956 with the CLAST-a and 0.965 with the CLAST-b. The Cronbach α of each subtest in the CLAST-a and CLAST-b ranged from 0.701 to 1.

#### Inter-rater reliability

The ICC among different raters was 1.

#### Equivalence between the CLAST-a and CLAST-b

The mean total score on the CLAST-a was 10.43 (10.60 on the CLAST-b) and the ICC between the two versions was 0.991. When the automatic speech item, which was identical in both versions, was removed, the ICC (0.989) did not change significantly. The ICC between parallel items in the two versions ranged from 0.786 to 0.983.

#### The relationship between educational levels and CLAST scores

The Mann-Whitney U test showed that the 163 subjects in the higher educational level group earned higher scores than the 88 ones in the lower-level education group. The difference was statistically significant (p_CLAST-a_ = 0.000<0.01, p_CLAST-b_ = 0.003<0.01).

**Fig 3:**
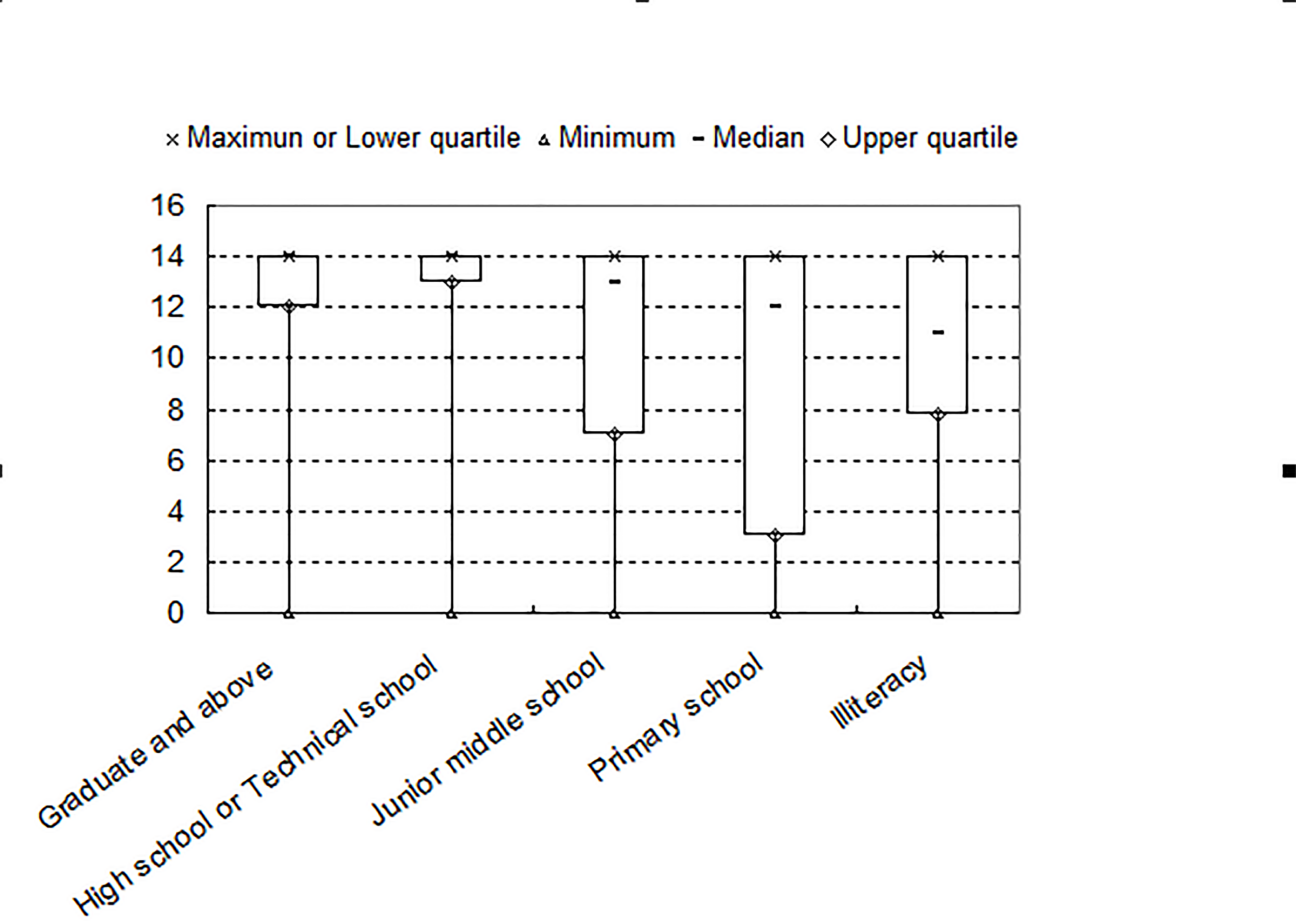
Box plot representing the total scores of the Chinese version of the Language Screening Test (CLAST-a/CLAST-b) in terms of educational levels. The maximum and the lower quartile of CLAST score were found to be identical.

Fig 3 shows the box plot representing the distribution of the total scores of CLAST-a and CLAST-b with regard to different educational levels(n = 261).

#### External validity

**Fig 4:**
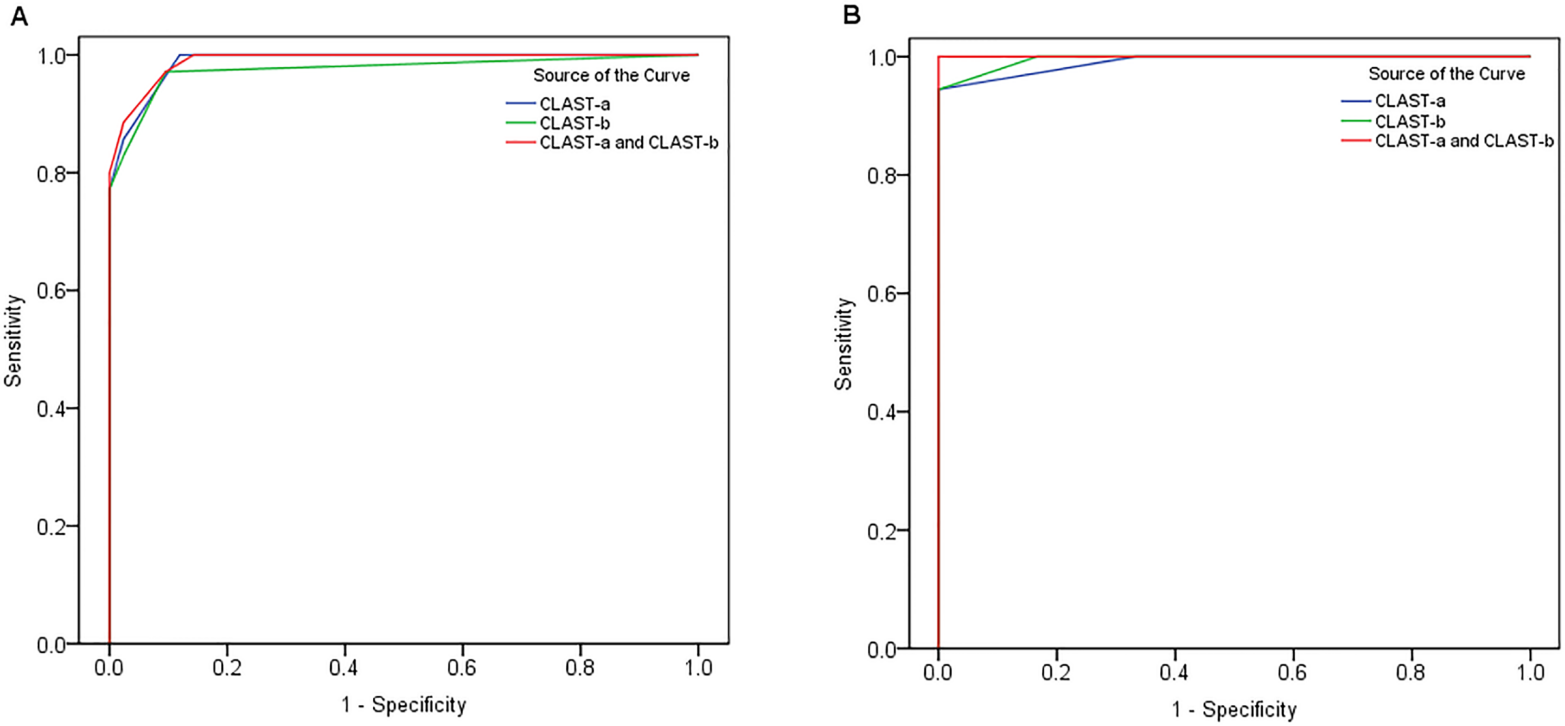
Receiver operating characteristic (ROC) curve of the Chinese version of the Language Screening Test (CLAST) in comparison with the Western Aphasia Battery (WAB) used for external validation. The blue, green, and red curves respectively represent CLAST-a, CLAST-b, add CLAST-a and CLAST-b combined. (A) In higher-level education group, at the point where CLAST-a score or CLAST-b score <14, specificity was 0.878 or 0.902 and sensitivity was 0.972 or 0.944. At the point where the combined score of CLAST-a and CLAST-b <27, the specificity was 0.902 and sensitivity was 0.944. (B) In lower-level education group, at the point where CLAST-a score or CLAST-b score <13, specificity of both (CLAST-a and CLAST-b) was 1 and sensitivity of both was 0.944. At the point where the combined score of CLAST-a and CLAST-b <26, both specificity and sensitivity were 1.

The diagnosis of aphasia can be establishedwhen the Aphasia Quotient (AQ) of WAB is <93.8 out of 100 [24]. The optimal cut-off point (score) of the CLAST varied with different educational levels (Fig 4A and 4B represents the patients with higher and lower educational levels, respectively).

**Fig 5:**
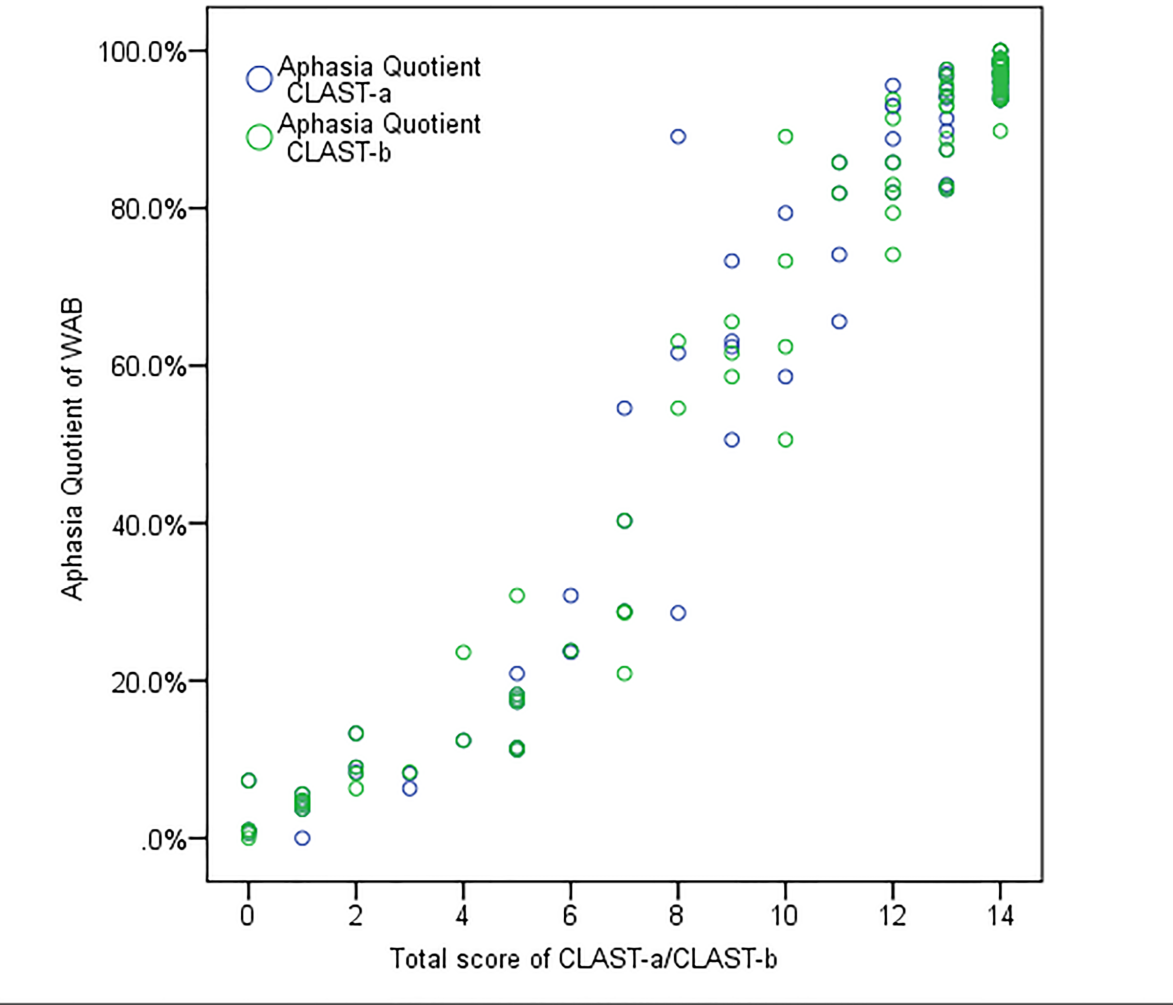
Scatter diagram between Aphasia quotient (AQ) of the Western Aphasia Battery (WAB) and total scores of the Chinese version of the Language Screening Test (CLAST-a/CLAST-b). The correlation between AQ of WAB and total score of CLAST-a/CLAST-b is represented by blue circles and green circles, respectively.

Fig 5 shows the correlations between the total score of the CLAST and WAB on a scatter diagram (Spearman coefficient between the CLAST and WAB was 0.920 (rho_CLAST-a_) or 0.921 (rho_CLAST-b_) (p<0.01).

**Fig 6:**
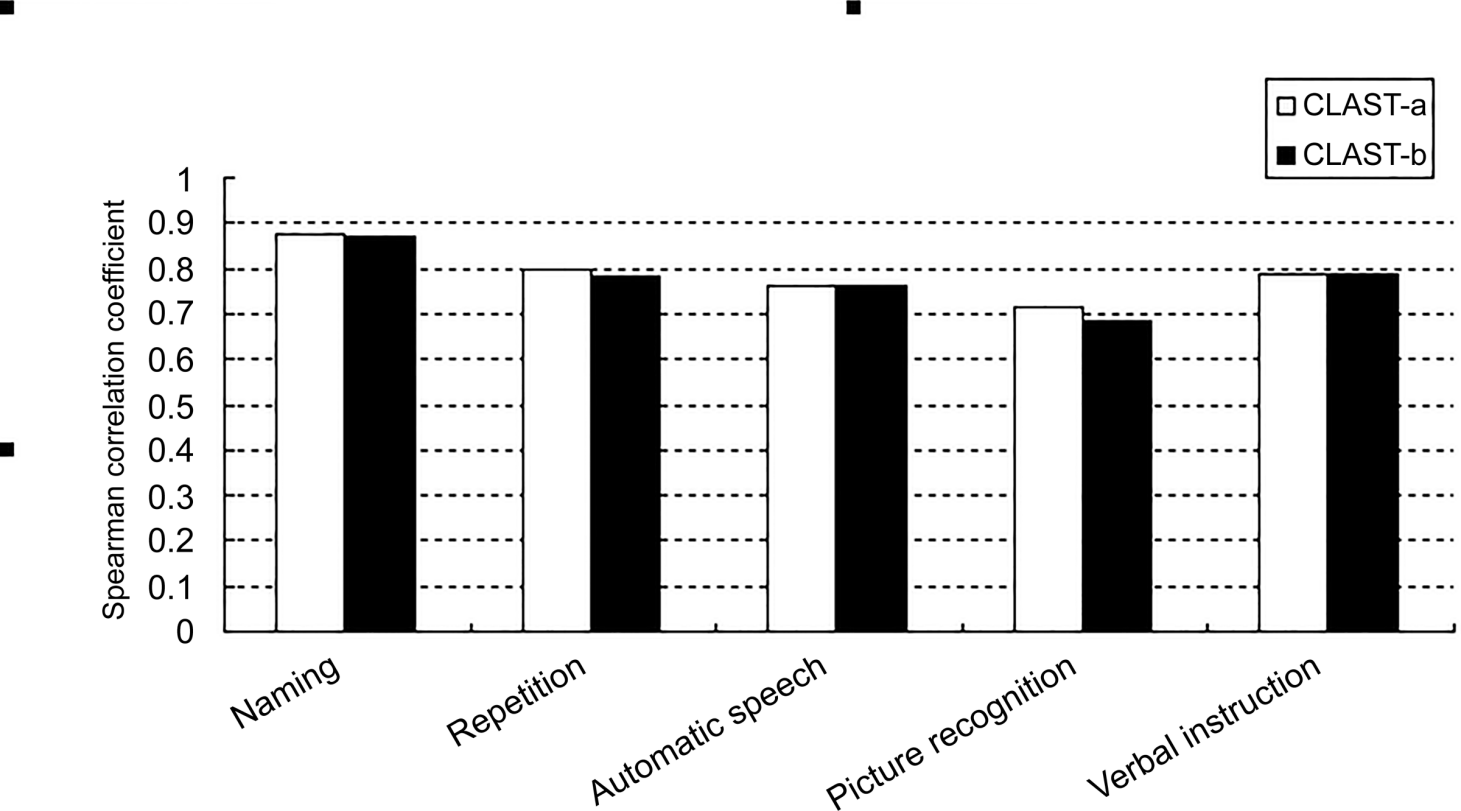
Histographic representation of the correlation between the Chinese version of the (CLAST) subtests and corresponding Western Aphasia Battery (WAB) items. The CLAST-a and CLAST-b are indicated by white and black bars, respectively. The coefficient of correlation between the CLAST and WAB ranged from 0.68 to 0.885 (p<0.01).

Fig 6 shows the correlation between the subtests of the CLAST-a and CLAST-b (with *rho* ranging from 0.68 to 0.885) on a histogram (*rho* was greater than 0.7 for all, except that correlation between “Picture recognition” of the CLAST-b and "Auditory comprehension" of the WAB was 0.68).

## Discussion

Our study showed that the final CLAST designed for screening aphasia in acute stroke patients was convenient and effective. The equivalence between the two versions of the CLAST was good. Both versions also had good inter-rater reliability, internal consistency, and validity. The external validity against the WAB was also good (the area under the ROC was 0.969 and the parallel items between the CLAST and WAB were well correlated). The median time to complete one initial version of CLAST was 98 seconds. The test could be carried out by an average medical physician. This CLAST is the first Chinese version of the LAST for quick screening of aphasia in acute stroke patients.

The CLAST has several advantages which are similar with LAST: it’s simple and easy to administer by any physician and avoids the impact of pre-existing illiteracy and hand paresis. Furthermore, the availability of two equivalent parallel versions minimizes possible retest effects.

The CLAST versus LAST: In the LAST study, either the LAST-a or LAST-b was used on a random basis and the data were pooled for analysis after the equivalence verification [9]. In this study, we tested the reliability and validity of each version separately (i.e., testing both versions of the CLAST in each single subject enrolled). When the two Chinese versions combined with each other, sensitivity and/or specificity may be further improved in clinical practice.

Compared with the LAST, the CLAST is different in a number of respects: (1) Patients with a higher-level education (junior middle school education and above) outdid their counterparts receiving lower-level education (only elementary school education and below) on the CLAST, suggesting that patients’ performance on the CLAST might be influenced by their educational background. Perhaps the items of CLAST we selected influence outcome, or the national cultural of China lead to language hierarchy in different educational background. (2) The ROC curve demonstrated that the external validity of the CLAST-a and CLAST-b was not as good as that of the LAST, and the optimal cut-off scores varied with different educational levels. In patients with lower educational levels, both sensitivity and specificity were 1 when the CLAST-a and CLAST-b were used in combination at the point of at which the total scores of CLAST-a and CLAST-b<26. Therefore, we suggest that to improve the screening accuracy in patients with lower educational levels, both versions of the CLAST should be used if conditions permit.

The present study had some limitations: Firstly, in our non-acute phase sample, patients were not included consecutively, and it was likely that the full spectrum of aphasia severity was not represented in the study cohort, which might increase the possibility of selection bias. Furthermore, the external validity was verified only in the stroke patients at non-acute phase because no generally-accepted diagnostic criteria for emergent aphasia were available. Finally, only the initial CLAST which had same item redundancy was administered in our enrolled patients, the final CLAST scores were obtained merely by “exchanging” and “deleting” scores from the initial CLAST in the enrolled patients, and the time to administer final CALST were beyond computation in our test. It might be better to re-validate the final CLAST in new groups.

The final CLAST detected a language deficit (when the CLAST-a and CLAST-b was <26 for patients with lower educational levels and the CLAST-a or CLAST-b was <14 for patients with higher educational levels) in 42.9% of the 154 patients admitted to our research centers, whereas aphasia was reported in only 17% to 38% of patients in other acute stroke series. The possible reasons might be: (1) the CLAST might have a higher sensitivity for aphasia; (2) early testing of the acute phase patients (within 72 hours after stroke onset), thus identifying patients who would go on to recover rapidly; (3) the acute patients had lower educational level on average and thus possibly higher yield rate. Identification of false-positive (nonaphasic) patients such as (a) patients with dysarthria; and (b) patients with initiative/executive dysfunctions (for example, the maximal response time of 5 seconds could penalize patients with initiative disorders); (c) in picture recognition subitems, the use of homophones with interwoven semantic relatedness as distractors might well increase the difficulty and then increase the false-positive rate, which might be more evident in patients with inadequate attention, and when the test was administered in patients who spoke dialectic versions of Mandarin.

In terms of educational levels, the maximum and the lower quartile of CLAST score were found to be identical.The possible reasons might be: (1)The number of non-aphasia patients (142 patients in total, composed by 54 patients referring by the WAB from the non-acute phase; and 88 patients, 57.1% from the 154 acute phase patients) out-numbered the aphasia ones (119 patients intotal, composed by 53 patients referring by the WAB from the non-acute phase; and 66 patients, 42.9% from the 154 acute phase patients); (2)Our research centers are general wards, and more patients with relatively minor to moderate stroke were enrolled (The median of NIHSS score was 3/5 in the acute/non-acute phase); (3) The sample size in terms of different educational levels might not be big enough, and the abnormal distribution of CLAST score might result;(4) CLAST may be deficient in discerning the aphasia severity.

To summarize, our study showed that the CLAST can be used at bedside and was proved to be a rapid and efficient tool for screening aphasia in patients with acute-phase stroke. Whether the CLAST has the potential for estimating the severity, evaluating outcome, or forecasting prognosis of aphasia in early post-stroke patients warrants further studies.

## Supporting information

S1 File Chinese version of the final CLAST and instructions of the final CLAST. (PDF)

S2 File English version of CLAST-a. (PDF)

S3 File English version of CLAST-b. (PDF)

S4 File The item redundancy details in initial CLAST. (DOCX)

S5 File The adjustment of redundant items in initial CLAST. (DOCX)

S6 File The comparsion of distractors between LAST and CLAST. (DOCX)

S7 File Data of stroke patients in the acute-phase. (XLSX)

S8 File Data of stroke patients in the non acute-phase. (XLSX)

S9 File Sources of data and methods of assessment (measurement). (DOCX)
