# Correction: A Chinese version of the Language Screening Test (CLAST) for early-stage stroke patients

**DOI:** 10.1371/journal.pone.0201938

**Published:** 2018-08-02

**Authors:** Hongyan Yang, Shenghua Tian, Constance Flamand-Roze, Ling Gao, Wei Zhang, Yan Li, Jiajia Wang, Zhou Sun, Ying Su, Libin Zhao, Zhihou Liang

There are errors in the author affiliations. The affiliations should appear as shown here: Hongyan Yang^1,#a^, Shenghua Tian^2^, Constance Flamand-Roze^3^, Ling Gao^1^, Wei Zhang^4^, Yan Li^5^, Jiajia Wang^6^, Zhou Sun^1^, Ying Su^1^, Libin Zhao^7^, Zhihou Liang^1^

**1** Department of Neurology, Union Hospital, Tongji Medical College, Huazhong University of Science and Technology, Wuhan 430022, China, **2** Department of Endocrinology, Union Hospital, Tongji Medical College, Huazhong University of Science and Technology, Wuhan 430022, China, **3** Department of Neurology, Centre Hospitalier du Sud Francilien, Corbeil-Essonnes 91100, France, **4** Department of Neurology, First Affiliated Hospital of Shanxi Medical University, Taiyuan 030001, China, **5** Department of Neurology, Luoyang Central Hospital affiliated to Zhengzhou University, Luoyang 471009, China, **6** Department of Neurology, Binzhou people’s Hospital, Binzhou 256610, China, **7** Department of Anesthesia, Maternal & Child Health Hospital of Bao’an District, Shenzhen 518101, China

**#a** Current Address: Department of Neurology, Longhua Branch of Shenzhen People’s Hospital, Shenzhen 518109, China
