# Supplementary material for: A Chinese version of the Language Screening Test (CLAST) for early-stage stroke patients
Source: PLoS One. 2018 May 4;13(5):e0196646. doi: 10.1371/journal.pone.0196646 (PMC5935384; doi:10.1371/journal.pone.0196646)
Supplement: S1 File — (PDF) [file pone.0196646.s001.pdf]

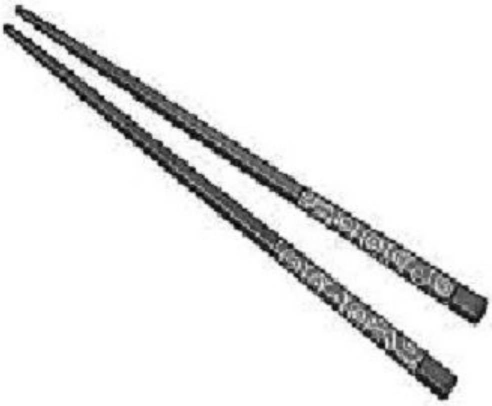

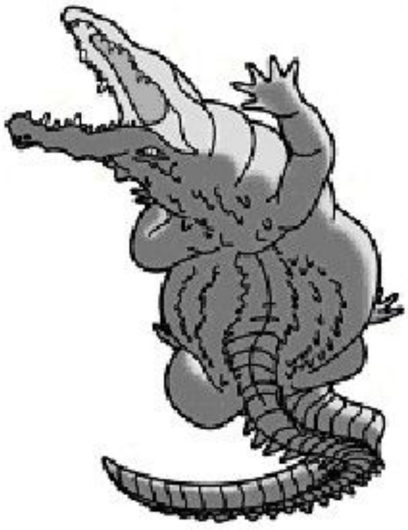

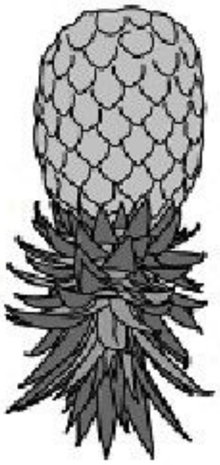

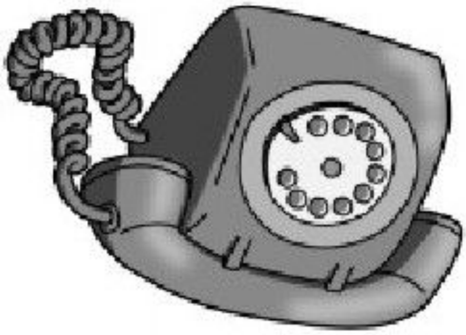

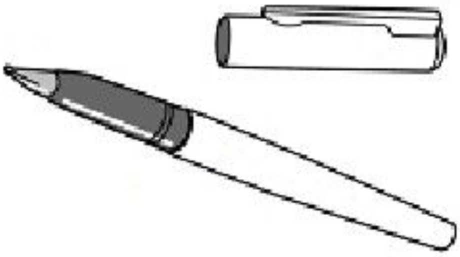

普通话版语言筛查量表
CLAST-a

姓名：
年龄：
性别：

教育程度：　　　　　　住院号：
诊断：
CT or MRI：

日期：___/___/___　　评定者：___

口语表达		分数	
命名	电话	/1	
	菠萝	/1	
	钢笔	/1	
	鳄鱼	/1	
	筷子	/1	
	命名分数		/5
复述	数学科学	/1	
	邮递员送了封信给我邻居	/1	
	复述分数		/2
自发言语	从一数到十	/1	
	自发言语分数		/1
口语表达分数			/8

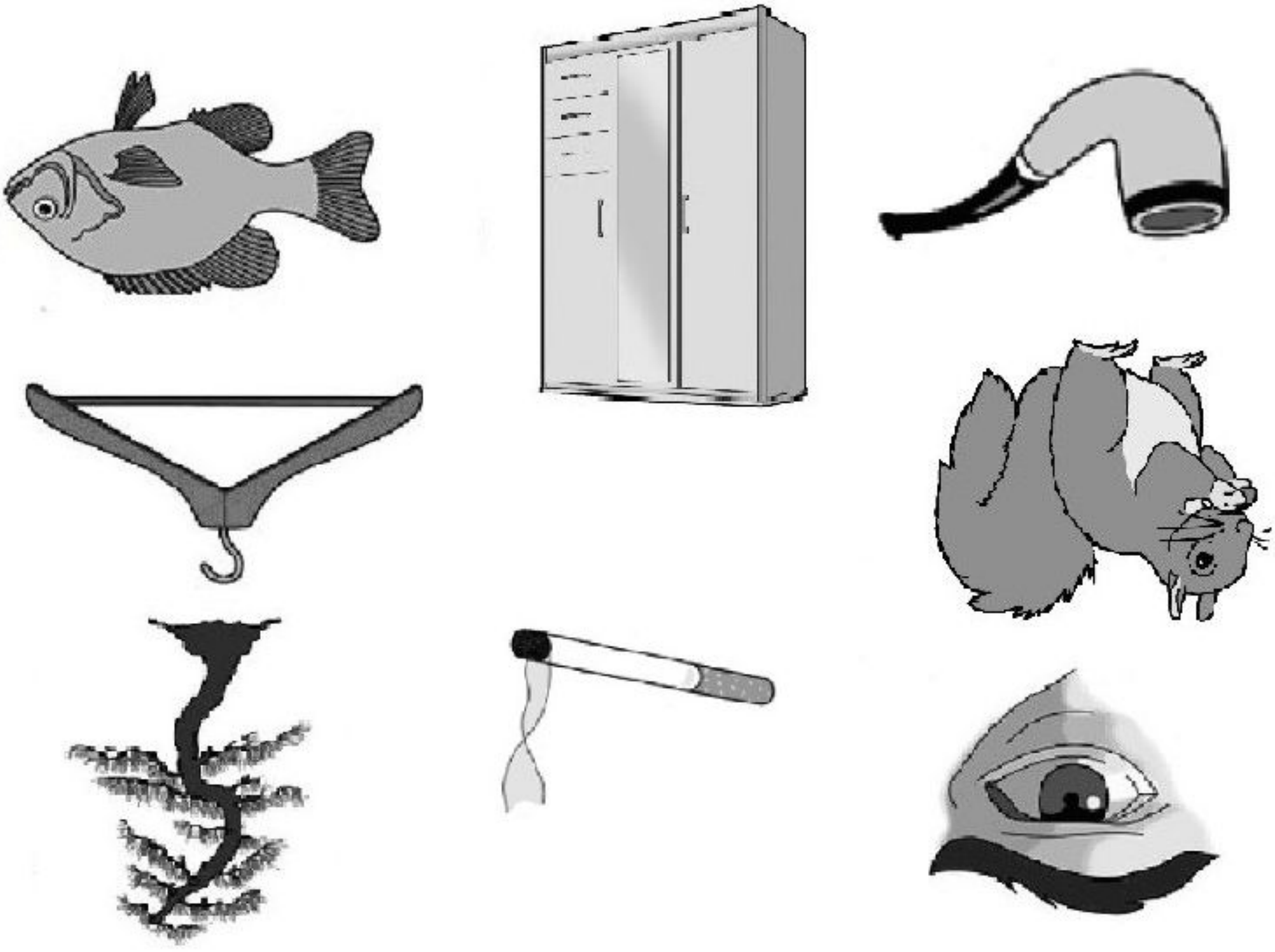

<table>
<tr><td colspan="2">言语理解</td><td colspan="2">分数</td></tr>
<tr><td rowspan="5">图片识别</td><td>松鼠</td><td>/1</td><td rowspan="4"></td></tr>
<tr><td>衣柜</td><td>/1</td></tr>
<tr><td>香烟</td><td>/1</td></tr>
<tr><td>眼睛</td><td>/1</td></tr>
<tr><td>图片识别分数</td><td></td><td>/4</td></tr>
<tr><td rowspan="3">言语执行</td><td>不要拿水杯而是拿钢笔</td><td>/1</td><td rowspan="2"></td></tr>
<tr><td>把你的一只手放在头发上，然后将一只手指放在鼻尖上</td><td>/1</td></tr>
<tr><td>言语执行分数</td><td></td><td>/2</td></tr>
<tr><td colspan="2">言语理解分数</td><td colspan="2">/6</td></tr>
<tr><td colspan="2">CLAST 总分</td><td colspan="2">/14</td></tr>
</table>

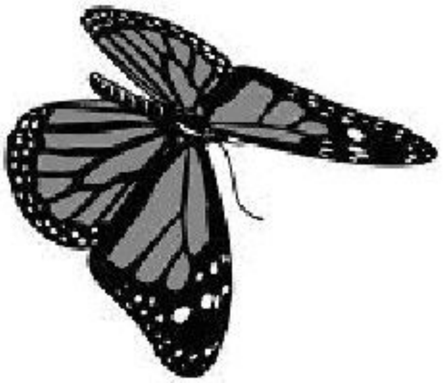

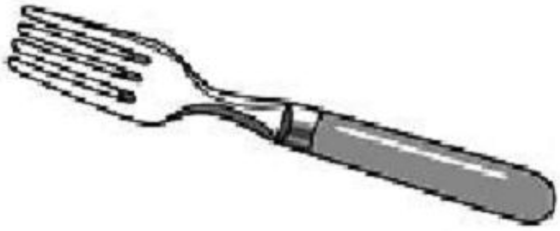

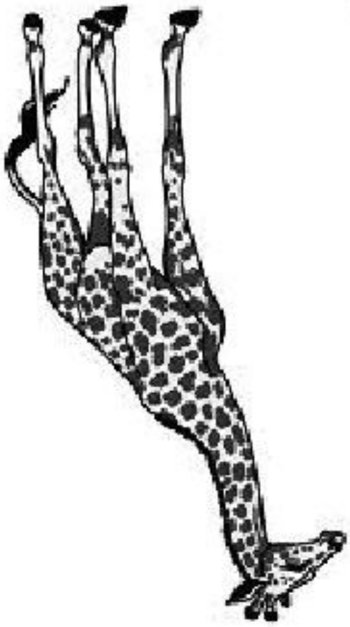

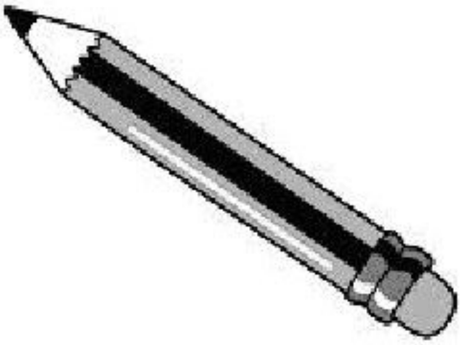

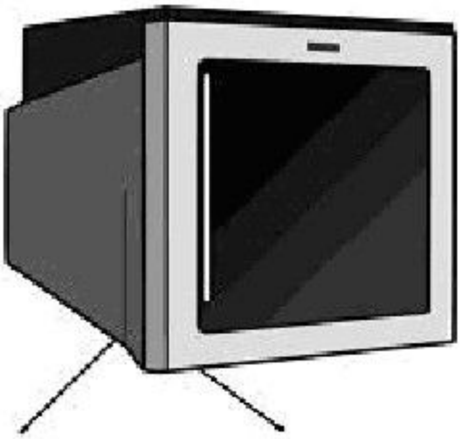

普通话版语言筛查量表
CLAST-b

姓名：
年龄：
性别：

教育程度：　　　　　　住院号：
诊断：
CT or MRI：

日期：___/__/__　评定者：___

口语表达		分数	
命名	铅笔	/1	
	电视机	/1	
	叉子	/1	
	长颈鹿	/1	
	蝴蝶	/1	
	命名分数		/5
复述	文学作品	/1	
	旅游者喜欢吃草莓冰淇淋	/1	
	复述分数		/2
自发言语	从一数到十	/1	
	自发言语分数		/1
口语表达分数			/8

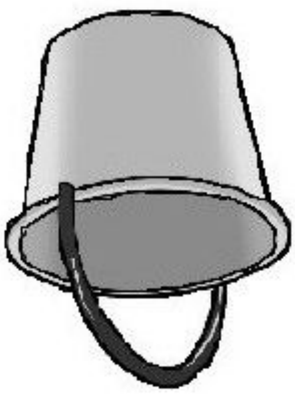
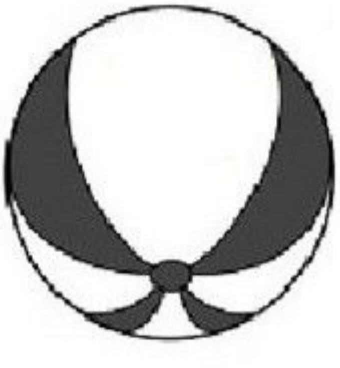
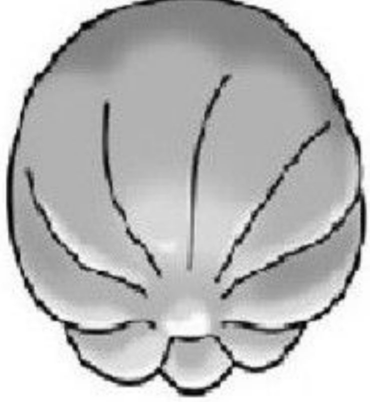
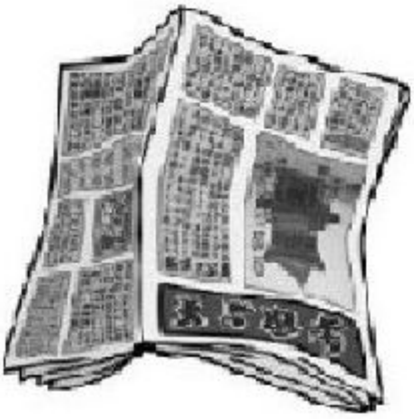
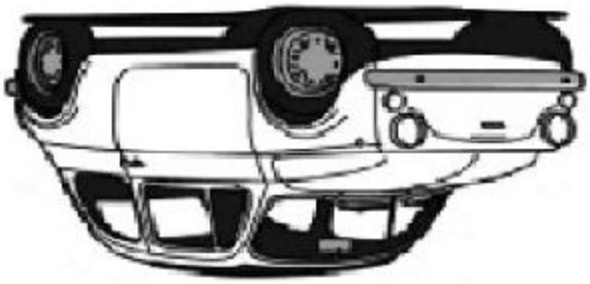
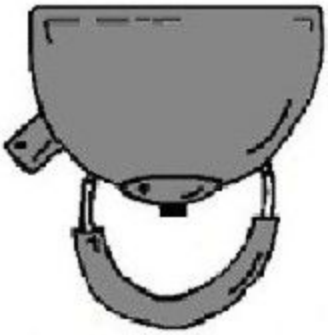
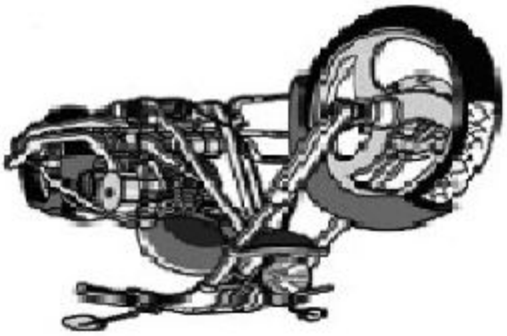
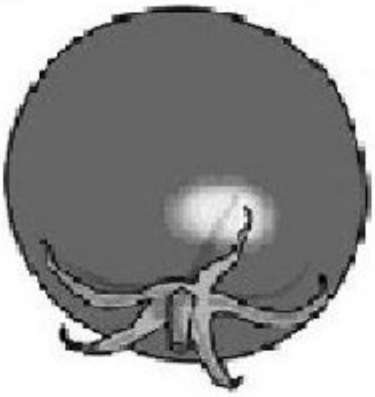

言语理解		分数	
图片识别	报纸	/1	
	水桶	/1	
	汽车	/1	
	西红柿	/1	
	图片识别分数		/4
言语执行	不要拿书本而是拿钥匙	/1	
	用你的一个手指去摸一边的耳朵，然后用两个手指去摸你的前额	/1	
	言语执行分数		/2
言语理解分数			/6
CLAST 总分			/14

Instructions of CLAST

Subjects were allowed 5 seconds to answer each item, and the answer was scored as either 1 (accurate answer) or 0 (false answer or failure to answer).

The assessment is administered according to the item orders without any hint.

If a subject gives a wrong answer and then makes correction on his or her own, the corrected answer will be accepted.

1. Naming:

Instructions: 《What is this?》

Responses using words synonymous with items on the test sheet are acceptable. For example, both "phone" and "telephone" are acceptable or correct answers. Responses indicating only the category a given item belongs to are not acceptable (For example: The answer "animal" is not an acceptable answer for item "giraffe".). The answers referring to a different species that belongs to the same genus with the item are not acceptable. For instance, "sika deer" is not an acceptable answer for the item "giraffe".

Items	*Acceptable (True)*	*Not acceptable (False)*
	CLAST-a	
Dianhua	*Dianhuaji*	*Shouji, yidongdianhua*
Bolo	*Fengli*	*Caomei*
Gangbi	*Moshuibi*	*Maobi, Yuanzhubi, Qianbi*
Eyu	*Alligator, cayman*	*Pangxie, Qingwa*
Kuaizi		*Shaozi, Chazi*
	CLAST-b	
Pencil		*Gangbi, Huabi, Yuanzhubi*
Dianshiji	*Dianshi, Heibaidianshiji*	*Diannao*
Fork		*Shaozi*
Changjilu		*Dongwu, Meihualu*
Hudie		*Fei e*

When an answer is unintelligible, 0 point will be awarded.

2. Repetition:

Instruction: 《Please repeat……》

Subjects are allowed to ask for repetition of a question by the examinee.

1 point will be awarded if a subject fully repeats a question or 0 point will be awarded if a subject fails to repeat the question correctly (including missing words, giving additional meaning, or using wrong word order).

3. Automatic speech:

Instruction: 《Count from 1 to 10.》

1 point is awarded if a subject 1 successfully counts the numbers) or 0 will be awarded if a subject fail to count the numbers, including omission, repetition of numbers or disarrangement of the number order)

4. Picture recognition:

Instructions: 《 Please point out……》

Any correction within 10 seconds is acceptable.

Examiner can give a second instruction if a subject fails to respond to the first instruction.

5. Verbal instructions:

Instructions:
Examiners should give the instructions strictly according to those listed in the test sheet.
1 point will be awarded if a subject correctly acts on the instructions. 0 point will be awarded if a subject fail to act on the instructions correctly or completely or in right order.
Examiner can give a second instruction if a subject fails to act on the first instruction.
