# Supplementary material for: A Chinese version of the Language Screening Test (CLAST) for early-stage stroke patients
Source: PLoS One. 2018 May 4;13(5):e0196646. doi: 10.1371/journal.pone.0196646 (PMC5935384; doi:10.1371/journal.pone.0196646)
Supplement: S2 File — (PDF) [file pone.0196646.s002.pdf]

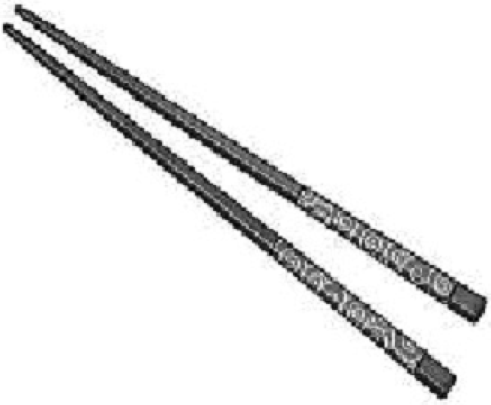

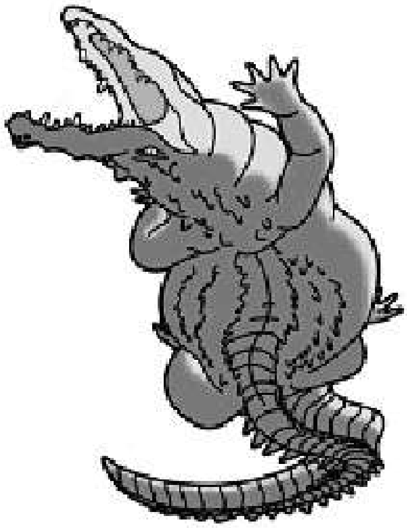

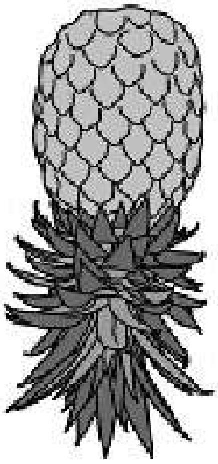

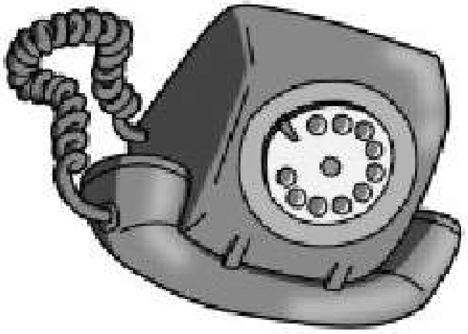

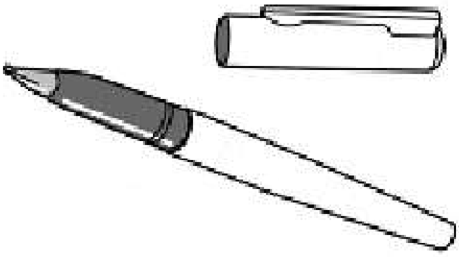

Chinese Version of Language Screening Test
CLAST-a

Name:
Age:
Gender:

Education: Admission number:
Diagnosis:
CT or MRI:

Date: ___/___/___ Rater: ___

Expression index		SCORE	
**Naming**	Phone	/1	
	Pineapple	/1	
	Pen	/1	
	Crocodile	/1	
	Chopsticks	/1	
	**Naming Score**		**/5**
Repetition	Mathematics	/1	
	The postman brings a letter to my neighbor	/1	
	**Repetition Score**		**/2**
**Automatic Speech**	Count from 1 to 10	/1	
	**Automatic speech Score**		**/1**
**Expression index Score**			**/8**

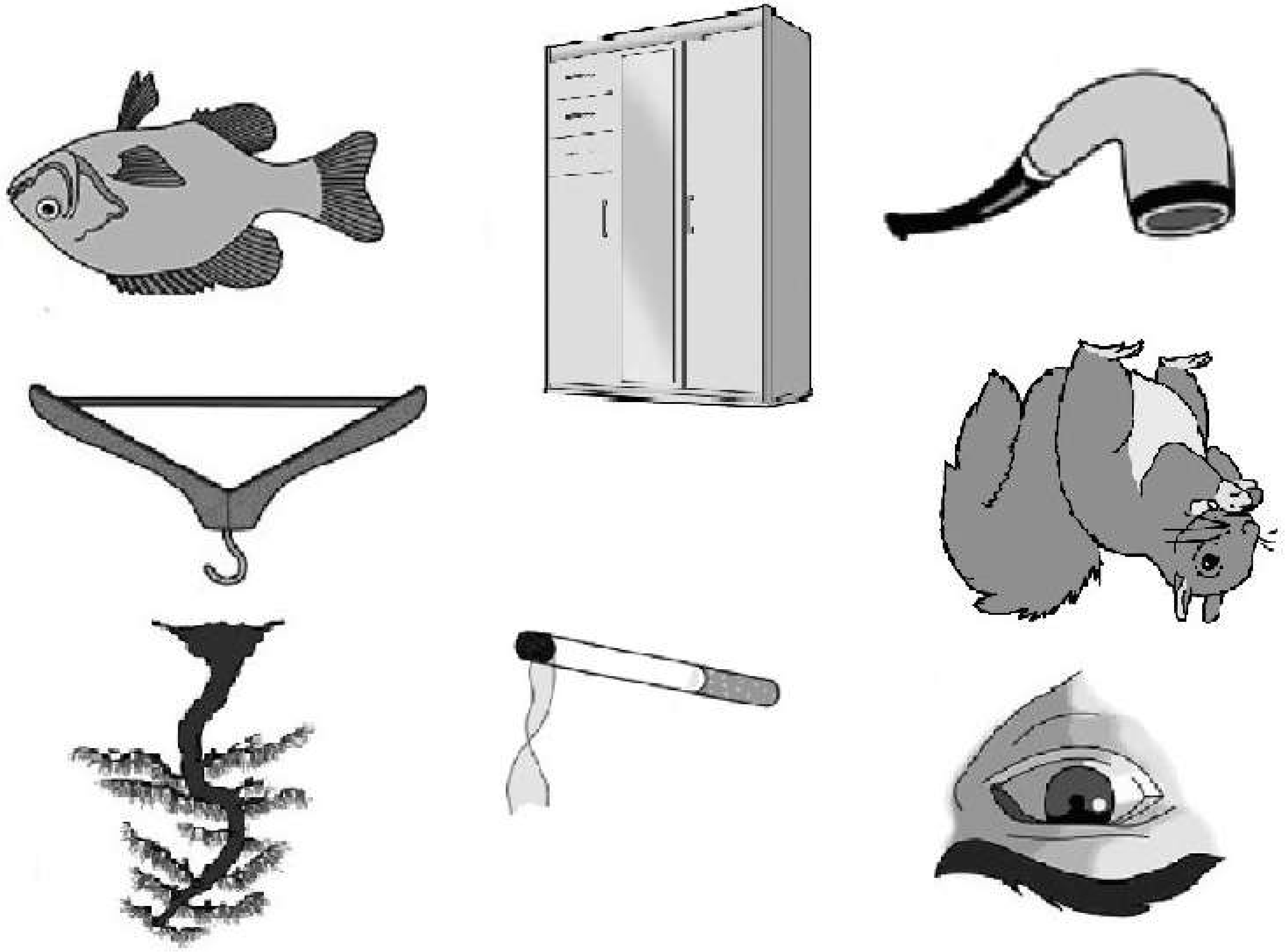

Receptive index		SCORE	
**Picture recognition**	Squirrel	/1	
	Wardrobe	/1	
	Cigarette	/1	
	Eye	/1	
	**Picture recognition Score**		/4
**Verbal instructions**	Don’t take the drink-glass but the pen	/1	
	Put a hand on your head, then a finger on your nose	/1	
	**Verbal instruction Score**		/2
**Receptive index Score**			/6
**CLAST TOTAL SCORE**		/14	
