# Supplementary material for: A Chinese version of the Language Screening Test (CLAST) for early-stage stroke patients
Source: PLoS One. 2018 May 4;13(5):e0196646. doi: 10.1371/journal.pone.0196646 (PMC5935384; doi:10.1371/journal.pone.0196646)
Supplement: S3 File — (PDF) [file pone.0196646.s003.pdf]

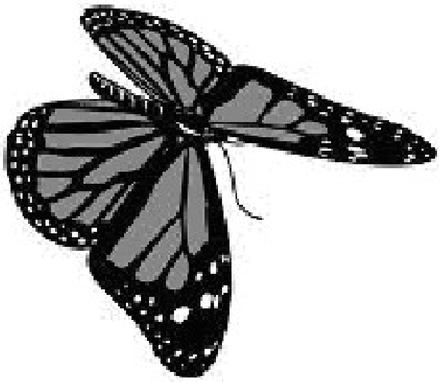
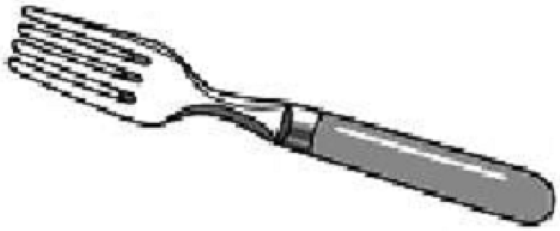
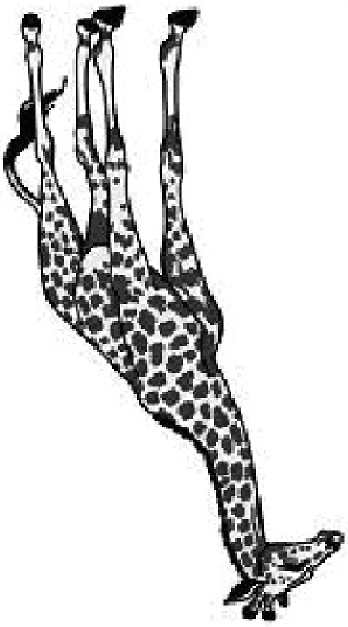
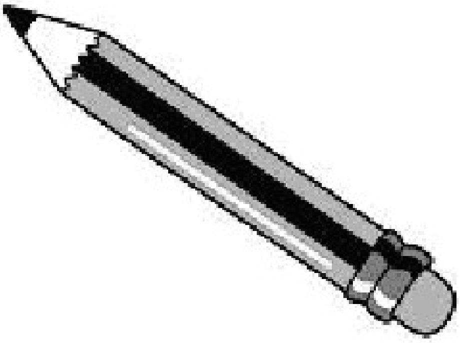
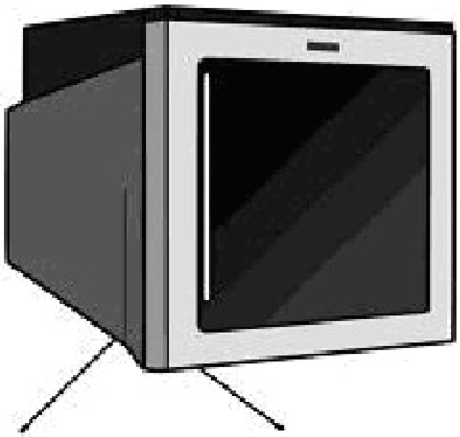

Chinese Version of Language Screening Test
CLAST-b

Name:
Age:
Gender:

Education: Admission number:
Diagnosis:
CT or MRI:

Date: ___/__/__ Rater: ___

**Expression index**		SCORE	
**Naming**	Pencil	/1	
	Television	/1	
	Fork	/1	
	Giraffe	/1	
	Butterfly	/1	
	**Naming Score**		**/5**
**Repetition**	Literature	/1	
	Vacationers would like strawberry ice-cream	/1	
	**Repetition Score**		**/2**
**Automatic Speech**	Count from 1 to 10	/1	
	**Automatic speech Score**		**/1**
**Expression index Score**			**/8**

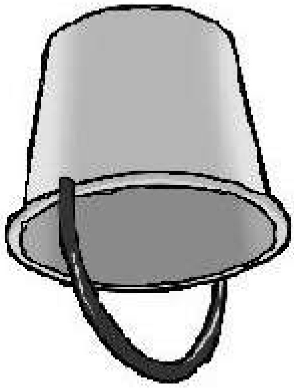
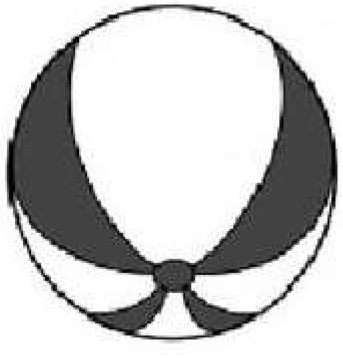
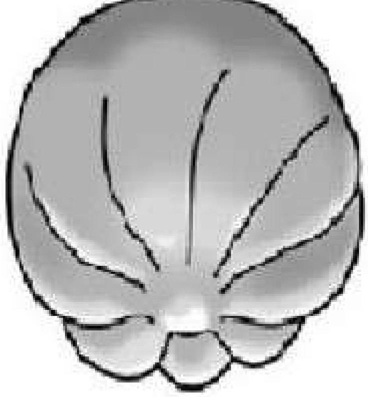
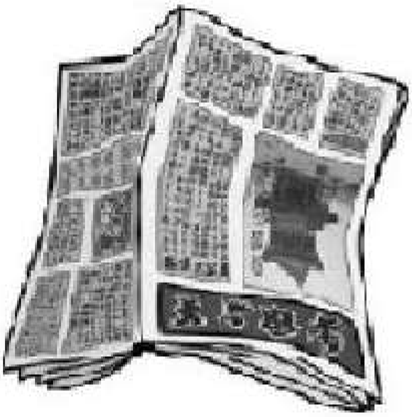
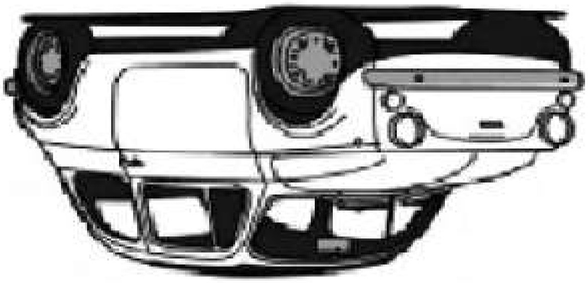
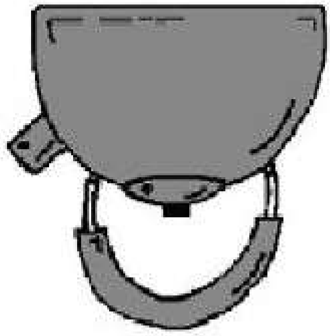
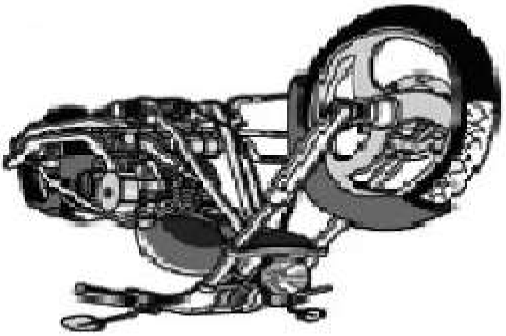
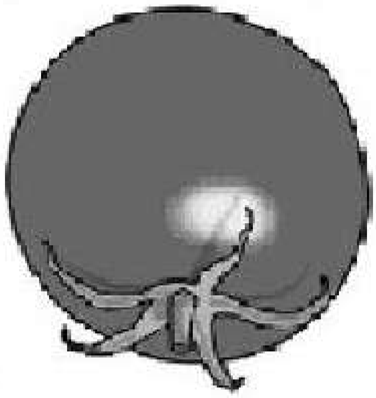

Receptive index		SCORE	
**Picture recognition**	Newspapers	/1	
	Bucket	/1	
	Car	/1	
	Tomato	/1	
	**Picture recognition Score**		/4
**Verbal instructions**	Don’t take the leaf but the key	/1	
	Touch one of your ears with one finger, then your forehead with two fingers	/1	
	**Verbal instruction Score**		/2
**Receptive index Score**		/6	
**CLAST TOTAL SCORE**		/14	
