# Supplementary material for: A Chinese version of the Language Screening Test (CLAST) for early-stage stroke patients
Source: PLoS One. 2018 May 4;13(5):e0196646. doi: 10.1371/journal.pone.0196646 (PMC5935384; doi:10.1371/journal.pone.0196646)
Supplement: S4 File — (DOCX) [file pone.0196646.s004.docx]

Item redundancy in the “Naming”

Between “Butterfly” and “Chopsticks”

None item redundancy

Item redundancy In the “Verbal instructions”

Between “Point at the ceiling” and “Don’t take the drink-glass but the pen”

CLAST-a

CLAST-b

Between “Point at the window” and “Don’t take the leaf but the key”

Item redundancy In the “Picture recognition”

Between “Knife” and “Pail”

Between “Cabbage” and “Eye”

The item redundancy details in initial CLAST(CLAST-a and CLAST-b) . The “Cabbage” in CLAST-a and the “Knife” in CLAST-b, with the anticipated to be reserves, were an extra pair of equivalent items that were added to the “Picture recognition” subtest when build the initial CLAST, but it was end up by discarding the reserves for item redundancy.
