# Supplementary material for: A Chinese version of the Language Screening Test (CLAST) for early-stage stroke patients
Source: PLoS One. 2018 May 4;13(5):e0196646. doi: 10.1371/journal.pone.0196646 (PMC5935384; doi:10.1371/journal.pone.0196646)
Supplement: S5 File — (DOCX) [file pone.0196646.s005.docx]

Exchanged with each other

“Fork” in naming subtest

“Chopsticks” in naming subtest

CLAST-a

CLAST-b

“Point at the window” in verbal instructions

“Point at the ceiling” in verbal instructions

Both were removed

“Knife” in picture recognition

Both were removed

“Cabbage” in picture recognition

The adjustment of items in initial CLAST to overcome item redundancy. After the above adjustment, the final CLAST(CLAST-a and CLAST-b) without item redundancy was obtained.
