# Supplementary material for: A Chinese version of the Language Screening Test (CLAST) for early-stage stroke patients
Source: PLoS One. 2018 May 4;13(5):e0196646. doi: 10.1371/journal.pone.0196646 (PMC5935384; doi:10.1371/journal.pone.0196646)
Supplement: S6 File — (DOCX) [file pone.0196646.s006.docx]

|  | Distracters | Disturbance type |  | Distracters | Disturbance type |
| --- | --- | --- | --- | --- | --- |
| Five items in the “Picture recognition” subtest in  **CLAST-a,**with 16 items in total. | “松鼠(Squirrel) and  “松树”(Pine) | Phonetic and semantic | Four items in the “Picture recognition” subtest in  **LAST-a,** with 15 items in total. | “Lapin”(Rabbit) and “Pin” (Pine) | Phonetic |
|  | “衣柜” (Wardrobe) and  “衣架” (Hanger) | Phonetic and semantic |  | “Cuillère” (Spoon) and “Lait caillé” (Cheese)” | Phonetic |
|  | “香烟”(Cigarette) and  “烟斗”(Tobacco pipe) | Phonetic and semantic |  | “Cigarette” (Cigarette) and  “Pipe”(tobacco pipe) | Semantic |
|  | “眼睛”（Eye） and  “鱼”(Fish) | visual |  | “Oeil”(Eye) and “Poisson”(fish) | Visual |
|  | *“白菜”(Cabbage) and  “萝卜”( Radish) | Semantic |  | | |
| Five items in the “Picture recognition” subtest in  **CLAST-b,**with 16 items in total. | “报纸”(Newspapers) and “包子” (Steamed stuffed bun)” | Phonetic and semantic | Four items in the “Picture recognition” subtest in  **LAST-b,** with 15 items in total. | “Chapeau” (Cap) and “Gateau” (Cake) | Phonetic |
|  | “水桶” (Bucket) and “水壶” (Kettle) | Phonetic and semantic |  | “Main” (Hand) and “Pain” (Bread) | Phonetic |
|  | “汽车”(Car) and  “摩托车”(Motorbike) | Semantic |  | “Voiture”(Car) and “Motocyclette”  (Motorbike) | Semantic |
|  | “西红柿”(Tomato) and  “皮球”(Rubber ball) | Visual |  | “Tomate”(Tomato)and “Balle”(Rubber ball) | Visual |
|  | *“菜刀”(Kitchen knife) and“锅铲”(Pancake turner) | Semantic |  | | |

*represent the extra pair of semantic distractors to the “Picture recognition” subtest in CLAST-a and CLAST-b. In the above distracters, the former ones are the correct answers, the latter ones are the corresponding interferences term of the former ones.
