# Supplementary material for: A Chinese version of the Language Screening Test (CLAST) for early-stage stroke patients
Source: PLoS One. 2018 May 4;13(5):e0196646. doi: 10.1371/journal.pone.0196646 (PMC5935384; doi:10.1371/journal.pone.0196646)
Supplement: S9 File — (DOCX) [file pone.0196646.s009.docx]

For each variable of interest, give sources of data and details of methods of assessment (measurement).

The equivalence between the two versions of the CLAST was assessed by calculating the intra-class correlation coefficient (ICC) from the two total scores and parallel items. Internal validity was assessed in terms of four aspects. First, the Spearman correlation matrix was used to detect item redundancy. Second, the ceiling or floor effect was detected. Third, construct validity was determined by utilizing factor analysis. Finally, the Mann-Whitney test was employed to compare the score of aphasia patients with their non-aphasia counterparts to verify discrimination validity. We computed Cronbach α to obtain the internal consistency. The ICC was used to represent inter-rater reliability. (Data source: S7 File. data of stroke patients in the acute-phase.)

The external validity was obtained by plotting the receiver operating characteristic (ROC) curve. (Data source: S8 File. data of stroke patients in the non acute-phase.)

The Mann-Whitney test was used to compare the performance of the two groups with different education levels to understand the relationship between CLAST scores and educational background. (Data source: S7 File. data of stroke patients in the acute-phase).
